# Clinico epidemiological profile of HIV - TB coinfection among PLHIV in coastal south India

**DOI:** 10.1186/1471-2334-14-S2-P32

**Published:** 2014-05-23

**Authors:** Unnikrishnan Bhaskaran

**Affiliations:** 1 Kasturba Medical College, Manipal University, Mangalore, India

## Background

Tuberculosis is the most common opportunistic infection diagnosed in HIV positive patients in India. HIV and Tuberculosis co-infection is a major public health problem as well as a leading cause of death in developing countries.

## Aims

To study the trends and clinico epidemiological profile of HIV and tuberculosis co-infection from January 2008 to December 2011.

## Method

A hospital based retrospective study was conducted on all subjects having HIV and Tuberculosis co-infection from January 2008 to December 2011. Data was collected using semi-structured proforma from Integrated Counselling and Testing Center (ICTC) records. All analysis was done using SPSS version 11.5. Statistical test Chi-square was done.

## Results

In 2011, 17.3% HIV positive cases were co-infected with tuberculosis in comparison to 6.5% in 2008, 14.9% in 2009 and 8.5% in 2010. HIV and Tuberculosis co-infection was more prevalent in males (69.3%) than in females (30.7%) and 90.9% of the study subjects were married. Most of the co-infected cases (89.8%) were on anti-tubercular treatment.

## Conclusion

HIV and Tuberculosis co-infection is under-diagnosed and under-treated. Thus there is a need to integrate Tuberculosis and HIV prevention programmes to face the threat of HIV associated tuberculosis.

